# Non-Targeted Detection of Synthetic Oligonucleotides in Equine Serum Using Liquid Chromatography–High-Resolution Mass Spectrometry

**DOI:** 10.3390/ijms25115752

**Published:** 2024-05-25

**Authors:** Emily Helmes, Jacob Montgomery, Gwendolyne Alarcio, Herra G. Mendoza, Jeffrey A. Blea, Peter A. Beal, Benjamin C. Moeller

**Affiliations:** 1 KL Maddy Equine Analytical Chemistry Laboratory, School of Veterinary Medicine, University of California, Davis, CA 95616, USAjmmontgomery@ucdavis.edu (J.M.); gsgonzales@ucdavis.edu (G.A.); 2 Department of Chemistry, University of California, Davis, CA 95616, USA; hdgrajo@ucdavis.edu (H.G.M.); pabeal@ucdavis.edu (P.A.B.); 3 School of Veterinary Medicine, University of California, Davis, CA 95616, USA; jablea@ucdavis.edu

**Keywords:** oligonucleotide, gene doping, liquid chromatography–mass spectrometry, serum, phosphorothioate

## Abstract

There is great concern in equine sport over the potential use of pharmaceutical agents capable of editing the genome or modifying the expression of gene products. Synthetic oligonucleotides are short, single-stranded polynucleotides that represent a class of agents capable of modifying gene expression products with a high potential for abuse in horseracing. As these substances are not covered by most routine anti-doping analytical approaches, they represent an entire class of compounds that are not readily detectable. The nucleotide sequence for each oligonucleotide is highly specific, which makes targeted analysis for these agents problematic. Accordingly, we have developed a non-targeted approach to detect the presence of specific product ions that are not naturally present in ribonucleic acids. Briefly, serum samples were extracted using solid-phase extraction with a mixed-mode cartridge following the disruption of protein interactions to isolate the oligonucleotides. Following the elution and concentration steps, chromatographic separation was achieved utilizing reversed-phase liquid chromatography. Following an introduction to a Thermo Q Exactive HF mass spectrometer using electrospray ionization, analytes were detected utilizing a combination of full-scan, parallel reaction monitoring and all ion fragmentation scan modes. The limits of detection were determined along with the accuracy, precision, stability, recovery, and matrix effects using a representative 13mer oligonucleotide. Following method optimization using the 13mer oligonucleotide, the method was applied to successfully detect the presence of specific product ions in three unique oligonucleotide sequences targeting equine-specific transcripts.

## 1. Introduction

In the past two decades, significant advances in therapies have emerged designed to modify DNA, impact the expression of genes, or alter the regulation of gene products for several disease states, including cancer, diabetes mellitus, and Duchenne muscular dystrophy [1]. While these technologies show significant potential in treating a variety of diseases or conditions, they represent new threats to racing safety and integrity efforts. Some proposed genetic targets for manipulation are focused on influencing muscle mass, recovery from injury, modifying the oxygen-carrying capacity, modifying the perception of pain, or affecting mental states by altering the expression of proteins associated with those pathways. Some of the likely protein targets are related to erythropoietin, insulin-like growth factor, growth hormone, myostatin, vascular endothelial growth factor, fibroblast growth factor, endorphin, and enkephalin [1,2]. Given the multitude of potential gene products that can be targeted, developing methodological approaches to identify the use of agents capable of altering these genes or their products is highly challenging.

**Figure 1:**
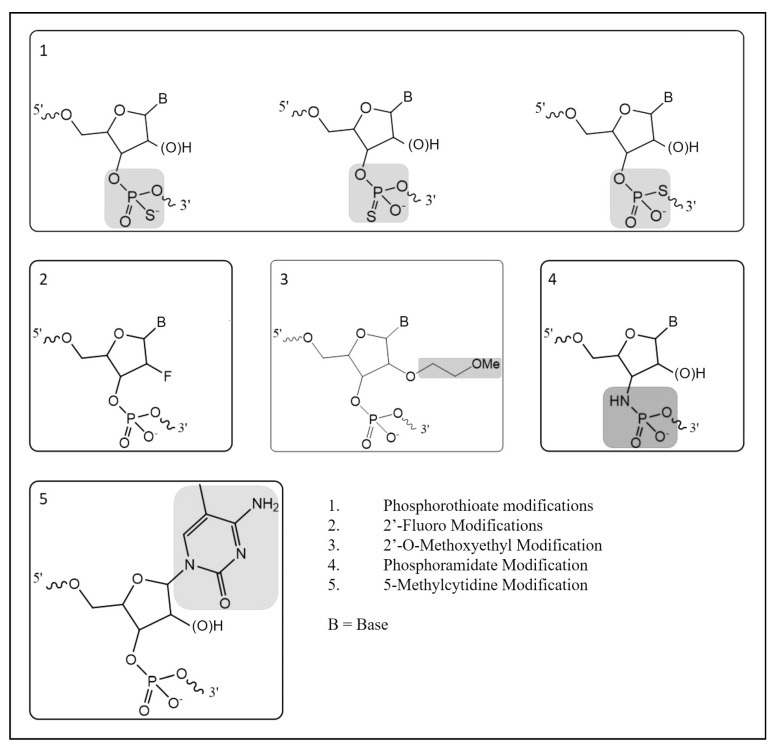
Common oligonucleotide modifications.

There are three main types of approaches used to manipulate the genome in gene doping: gene silencing, gene transfer, and gene editing [2]. Many gene-silencing technologies utilize heavily modified versions of RNA to interfere with gene expression and these modifications are performed to improve the stability, pharmacokinetics, pharmacodynamics, and target sequence recognition functionality [3]. Due to the rapid degradation of RNA molecules in biological matrices, synthetic modifications have been made with the aim of increasing stability by increasing resistance to exo- or endonucleases. In turn, the presence of more than ten phosphorothioate modifications in an oligonucleotide result in decreased nuclease activities, allowing the molecule to reach their targets without significant degradation [4]. Further modifications have been developed to improve pharmacokinetics and pharmacodynamics. The five of the most common oligonucleotide modifications include phosphorothioate, 2′-fluoro, 2′-O-methoxyethyl, phosphoramidate, and 5-methylcytidine (Figure 1) [4,5]. While there are several non-natural modifications that have been developed, the use of phosphorothioate modifications is commonly found in most antisense oligonucleotides (ASOs) and small interfering RNA (siRNA) molecules under development. Because the phosphorothioate modification is not naturally occurring in high abundance, this creates an opportunity to develop methodologies capable of detecting their presence in biological matrices for racehorses and other performance athletes where the use of these technologies is prohibited.

There are many technologies available to detect modified oligonucleotides in biological matrices, including mass spectrometry, matrix-assisted laser desorption ionization-time-of-flight mass spectrometry (MALDI-TOF-MS), liquid chromatography–electrospray ionization–mass spectrometry (LC-ESI-MS), or polymerase chain reaction (PCR)-based approaches [1,2,3,6,7,8,9]. Previously, phosphorothioated oligonucleotides were analyzed using MALDI-TOF-MS to determine their sequences using the combination of the observed precursor and product ions and extensive post-acquisition analysis [9]. Additionally, the product ion of the phosphorothioate moiety, 94.9362 *m*/*z*, has been successfully detected using quadrupole-time-of-flight tandem mass spectrometry for the targeted and non-targeted analysis of phosphorothioated oligonucleotides spiked in horse plasma [6]. With oligonucleotides having a high molecular weight (>3500 Da), it is important to have an instrument with high mass accuracy and sufficient mass resolution to resolve potential interferences and improve sensitivity and selectivity. Accordingly, a non-targeted LC-MS-based analytical method was developed to target the exogenous phosphorothioate modifications present in the ASOs and siRNA in equine serum.

## 2. Results

Over multiple analyses, the limits of detection were determined along with the accuracy, precision, stability, recovery, and matrix effects using a representative 13mer oligonucleotide (PS-1). Following method optimization using the 13mer oligonucleotide, the method successfully detected the presence of the phosphorothioate product ion in three unique oligonucleotide sequences. Two of the sequences targeted equine-specific transcripts for myostatin and hypoxia-inducible factor prolyl hydroxylase.

### 2.1. Linearity and Limits of Detection and Quantitation

**Figure 2:**
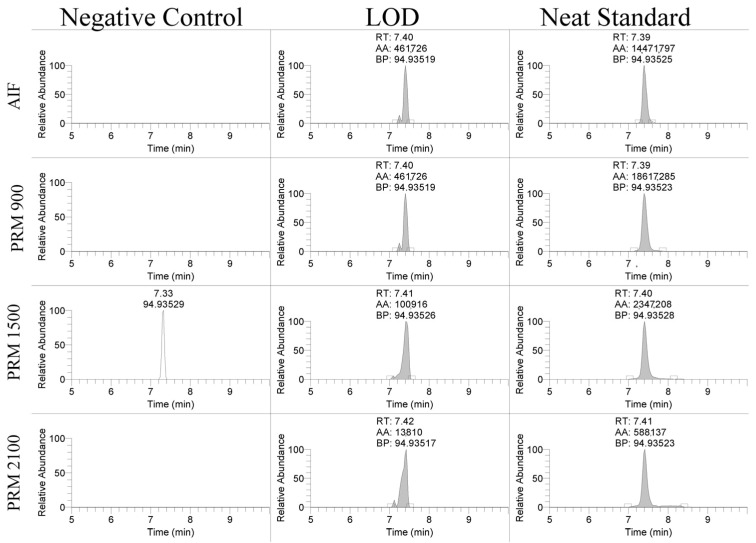
Representative extracted ion chromatograms of 94.935 *m*/*z* product ion using AIF and PRM scan modes in negative control serum, spiked serum at the limit of detection, and a neat standard.

The linearity of the assay was assessed over three days of validation and overall had regression coefficients > 0.99. The limit of detection (LOD) and limit of quantitation (LOQ) for the PS-1 oligonucleotide were assessed using different scan types. The product ion, 94.935 *m*/*z*, was detectable between 10 and 50 ng/mL using the AIF and PRM scan modes shown in Figure 2. The LOD was 25 ng/mL for the PRM-900 *m*/*z* scan, 10 ng/mL for the PRM-1500 *m*/*z* scan, 10 ng/mL for the PRM-2100 *m*/*z* scan, and 50 ng/mL for the AIF scan. The LOQ was 50 ng/mL for the PRM-900 *m*/*z* scan, 25 ng/mL for the PRM-1500 *m*/*z* scan, 50 ng/mL for the PRM-2100 *m*/*z* scan, and 50 ng/mL for the AIF scan. Similar results were observed for the EGL9 and MYO with limits of detection of 50 and 100 ng/mL, respectively. These detection limits are in line with reports in the ~70–100 ng/mL range using MALDI-TOF MS and LC/ESI-QTOF [6,9].

### 2.2. Accuracy, Precision, and Stability

The inter-day accuracy and precision were assessed by calculating the percent difference and relative standard deviation across three days of validation, showing adequate results (Table 1). These data indicate that while the methodology may not be an approach routinely utilized in most racing laboratories, the extraction and detection approaches are sufficiently reproducible for the qualitative identification for a batch of samples. 

As shown in Table 2, the stability was assessed at 25 °C for 1 day, 2–8 °C for 1 and 27 days, and at −20 °C for 27 and 28 days, assessing one and two freeze/thaw cycles. These results suggest that extended storage of collected serum samples at either room temperature or between 2 and 8 °C should be avoided due to the high potential for degradation and false negatives. The data suggest that storage at −20 °C with freeze/thaw cycles provides an adequate stability to allow for an analysis within the sample collection, transit, and storage conditions commonly utilized in racing chemistry. 

### 2.3. Method Application

**Figure 3:**
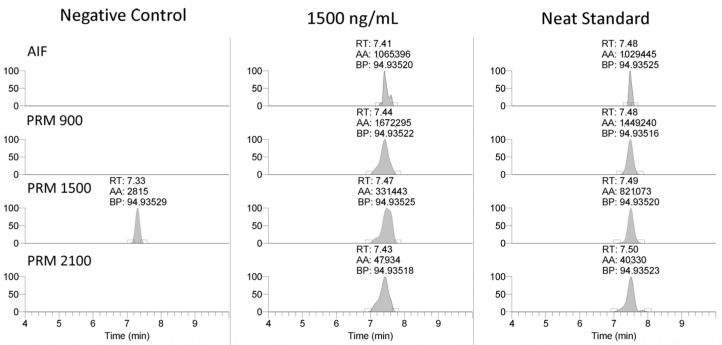
Representative extracted ion chromatograms (94.935 *m*/*z*) showing application to Beal oligonucleotide.

**Figure 4:**
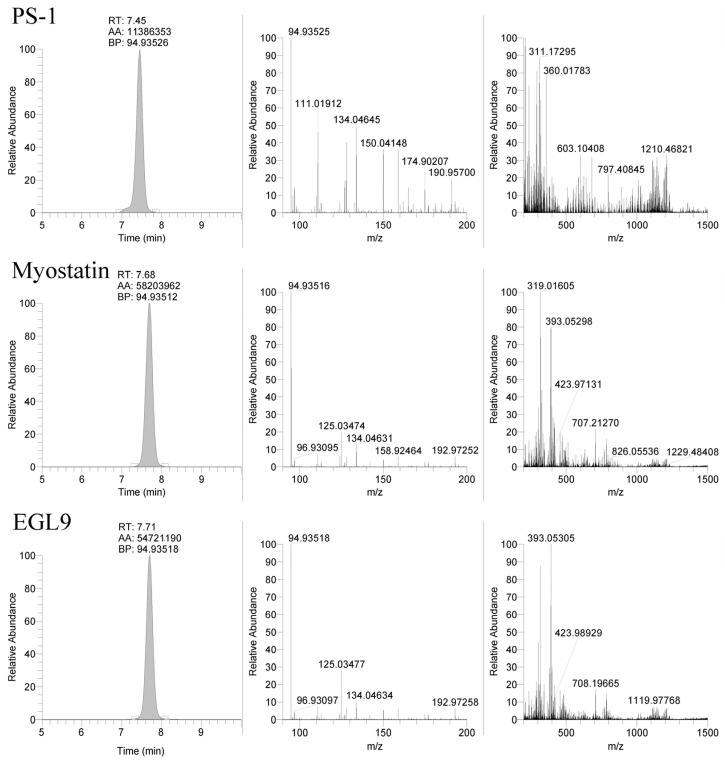
Representative extracted ion chromatograms (94.935 *m*/*z*) and MS2 spectra from 1500 *m*/*z* with 601 *m*/*z* scan window for PS-1, MYO, and EGL9 oligonucleotides.

**Figure 5:**
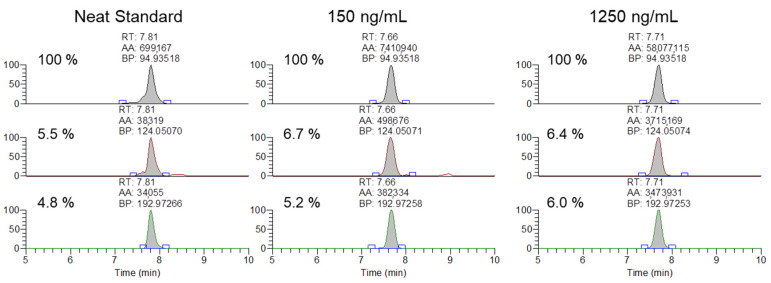
Representative extracted ion chromatograms and ion ratios for the 94.935, 124.050, 192,972 *m*/*z* product ions from 1500 *m*/*z* with 601 *m*/*z* scan window for MYO oligonucleotides in a neat standard, and serum samples spiked at 150 and 1250 ng/mL.

**Figure 6:**
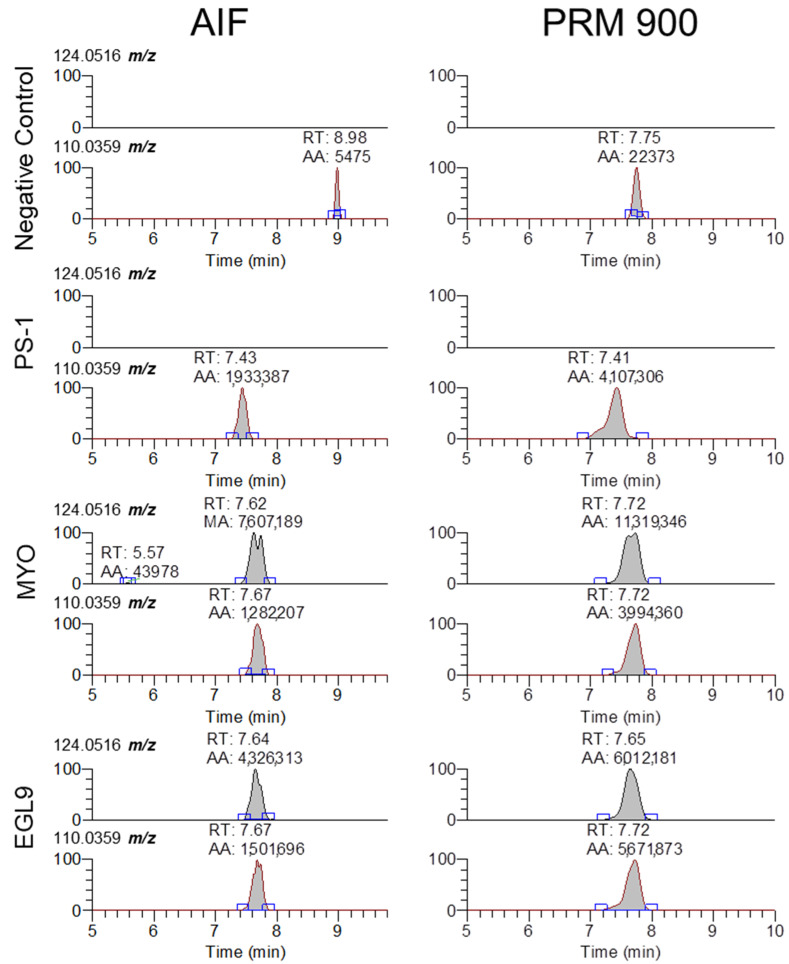
Representative extracted ion chromatograms for 5-methyl-cytidine and cytidine (124.0516 and 110.0359 *m*/*z*) from MS2 scans using AIF and 900 *m*/*z* with 601 *m*/*z* scan window for negative control serum and serum fortified with the PS-1, MYO, and EGL9 oligonucleotides at the high QC level.

**Figure 7:**
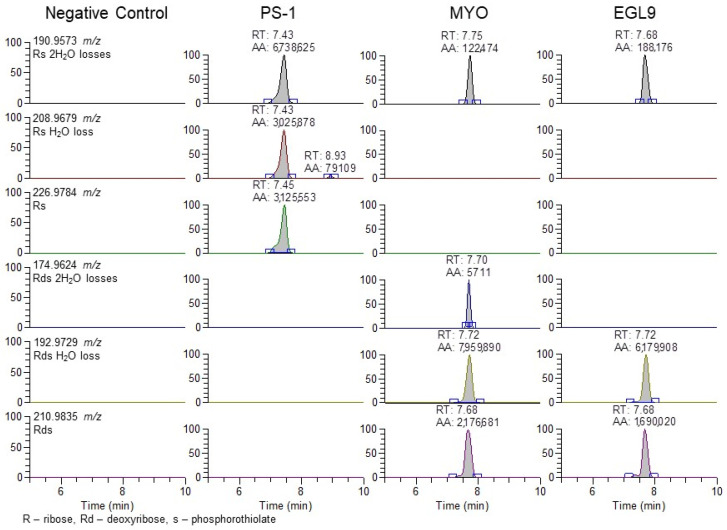
Representative extracted ion chromatograms for PRM 900 *m*/*z* with 601 *m*/*z* scan window for negative control serum and serum fortified with the PS-1, MYO, and EGL9 oligonucleotides at the high QC level.

The LC-MS method was applied to the equine-specific transcripts for myostatin and hypoxia-inducible factor prolyl hydroxylase (MYO and EGL9), as well as a non-commercially synthesized oligonucleotide (Beal), with the sequence shown in Table 3. The method successfully detected the MYO and EGL9 oligonucleotides, as well as the Beal oligonucleotide with only four phosphorothioate modifications present (Figure 3 and Figure 4). The presence of additional unique product ions was investigated using Ariadne software (https://ariadne.riken.jp/ modified 2 September 2023) to generate theoretical accurate masses [10]. As shown in Figure 5, Figure 6 and Figure 7, additional product ions focused on either phosphorothioate modifications or modified bases were observable. 

After the initial validation of the methodology was conducted, the method was applied to analyze 20 previously cleared serum samples submitted by regulatory clients, with the extraction and analysis completed within a day of initiation. None of the analyzed samples contained any of the observed exogenous modifications. Finally, the ability of the methodology to meet the AORC guidelines for identification by chromatography and mass spectrometry was assessed using samples spiked with the MYO oligonucleotide at 150 and 1250 ng/mL. As shown in Figure 5, the samples fortified at the low and high QC levels were readily confirmable using the 94.935, 124.050, and 192.972 *m*/*z* product ions. 

## 3. Discussion

### 3.1. Optimization of Liquid Chromatography and Mass Spectrometry

The selection of the mobile phase solvents and modifiers is critical for good chromatographic performance and high sensitivity using mass spectrometry. Previous studies found that using fluorinated alcohols as an acidic modifier in the mobile phase increased the signal intensity greatly when paired with hydrophobic ion-pairing agents such as DIEA, an alkylamine ion-pairing agent [11,12,13]. To increase the signal, it was found that pairing HFIP with DIEA resulted in a dramatic increase in ionization efficiency as compared to only DIEA as a mobile phase modifier due to improved thermodynamics for gas phase ionization [13]. DIEA retains the oligonucleotides much better than less hydrophobic ion-pairing agents because it has a higher tendency to bind with the C18 or similar stationary phase. The use of HFIP and DIEA mobile phase modifiers with a reverse phase column allowed for good retention of the targeted oligonucleotides with the retention times for the PS-1, MYO, and EGL9 oligonucleotides eluting over a one-minute window between 7 and 8 min as shown in Figure 4. The gradient compositions allow for the compounds to be highly retained, and elution from the stationary phase required a high composition of organic solvent, replicating the results observed by Basiri and colleagues [11]. Further improvements in chromatographic separation and performance may be achievable using a more specialized stationary phase and/or one with a smaller particle size and a more modern chromatographic system. 

The analysis of oligonucleotides is challenging due to their large size, complex composition, and physiochemical properties that differ from most analytes commonly targeted in drug testing laboratories [3]. The majority of the previous work has focused on the targeted detection of known oligonucleotides, but recent studies targeting the phosphorothioate product ion (94.935 *m*/*z*) allowed for high sensitivity and selectivity for oligonucleotides containing this modification [6,14]. Based upon these studies, our methodology was optimized for the mass detection of the 94.935 *m*/*z* product ion using both AIF and PRM scan modes. The collision energy for the AIF scan spectra was optimized to have an in-source CID of 60 eV to aid in the in-source fragmentation of mobile phase modifiers that were observed with high intensity. PRM scans with large precursor ion windows (601 *m*/*z*) allow for an overlapping of precursor mass ranges from 600 to 2400 *m*/*z*, which allows for the isolation of multiple different charge states that are commonly observed for oligonucleotides. PRM acquisition above 2400 *m*/*z* was not possible due to the high mass cutoff of the quadrupole. The use of stepped collision energy was utilized to balance the acquisition with the highest sensitivity (ce 40) for the phosphorothioate product ion with the generation of additional product ion spectra with lower collision energy (ce 20). While the stepped collision energy does allow for the observation of a large number of product ions, the use of the ce at 40 will result in more fragmentation and a reduction in the ability to have more sequence-specific product ions. As the targeted oligonucleotides are ionized as a mixture of multiply charged species, the use of the AIF or PRM scans resulted in the fragmentation of many species simultaneously. This makes interpretation spectra to identify sequence-specific fragmentation (a, b, c, y, and z—series ions) challenging to accomplish. Finally, full-scan SIM from 600 to 6000 *m*/*z* allows for the evaluation of precursor ions that may be present at the same retention time as the suspect finding observed for the 94.935 *m*/*z* product ion, which can allow for the subsequent targeted acquisition using a PRM scan with a small mass window to acquire high-quality MS/MS spectra. 

While our approach and the validation data presented in this approach are focused on the detection of the 94.935 *m*/*z* product ion, the fragmentation-rich spectra generated via HCD collision offer additional opportunities to target other non-natural modifications present in oligonucleotides that can be successfully extracted and analyzed using our approach. The fragmentation of oligonucleotides in the gas phase has been investigated in great detail for over 30 years [15]. As there are a large number of natural and non-natural modifications to either the linker, sugar, or base components of a natural or synthetic oligonucleotide, the interpretation of the resulting MS/MS spectra and correct assignment of the sequence without prior knowledge is highly complex, though there are a number of software platforms available to aid in the interpretation [9,10]. While the detection of the presence of the 94.935 *m*/*z* product ion may be sufficient for an initial screening approach, based upon the Association of Official Racing Chemist guidelines for identification using mass spectrometry, the use of a single ion for confirmation analysis is not suitable for a confirmatory analysis [16]. In review of our MS/MS spectra, there are a large number of product ions that can be selected for evaluation, though without prior knowledge of the targeted sequence corelating the specific product ions to a sequence is highly challenging. While fragmentation along with an oligonucleotide sequence generates a large number of product ions that can comprise one or more nucleosides, the use of software such as Ariadne (https://ariadne.riken.jp/ modified 2 September 2023) or those provided by instrument vendors can facilitate the identification of additional products that can be used for identification purposes [10]. The determination of an oligonucleotide sequence from unknown samples will likely require targeted methodologies with lower collision energies capable of generating high-quality fragmentation. One example is using the presence of a modified base such as 5-methyl-cyitdine using the 124.0516 *m*/*z* ion and comparing the signal ratio to that of the unmodified cytidine 110.0359 *m*/*z*. As seen in Figure 6, the 5mC-specific product ion is only observed in serum samples fortified with either the MYO or EGL9 oligonucleotides, while cytidine is observable in the samples fortified with the PSM, MYO, and EGL9 oligonucleotides. As 5mC is an endogenous modified base found in both RNA and DNA at low levels, further investigation is warranted though its presence in conjunction with other rare or non-naturally occurring modifications may indicate the presence of a prohibited synthetic oligonucleotide [17]. 

The use of the 94.935 *m*/*z* product ion is highly diagnostic for the presence of the phosphorothioate in the oligo sequence; though, in review of our product, for the ion spectra shown in Figure 4 in addition to the readily observable product ions associated with the bases for cytidine (110.0359 *m*/*z*), thymidine (125.0356 *m*/*z*), adenine (134.0472 *m*/*z*), and guanine (150.0421 *m*/*z*), additional 190.9573 and 192.9729 *m*/*z* product ions are observable in the PS1 and MYO/EGL9 spectra. As shown in Figure 7, these fragments are associated with the product ions of the ribose sugar with the phosphorothioate with either a single (192.9729 *m*/*z*—deoxyribose) or double (190.9573 *m*/*z*—ribose) loss of water. Additional ions corresponding to either the water losses or the intact product ions are shown in Figure 7. The PS1 oligo utilizes a fully phosphorothiolated backbone containing only ribose sugars, while the MYO and EGL are fully phosphorothiolated containing a mixture of deoxyribose sugars and 2′-methoxyethoxy-modified sugars. 

**Figure 8:**
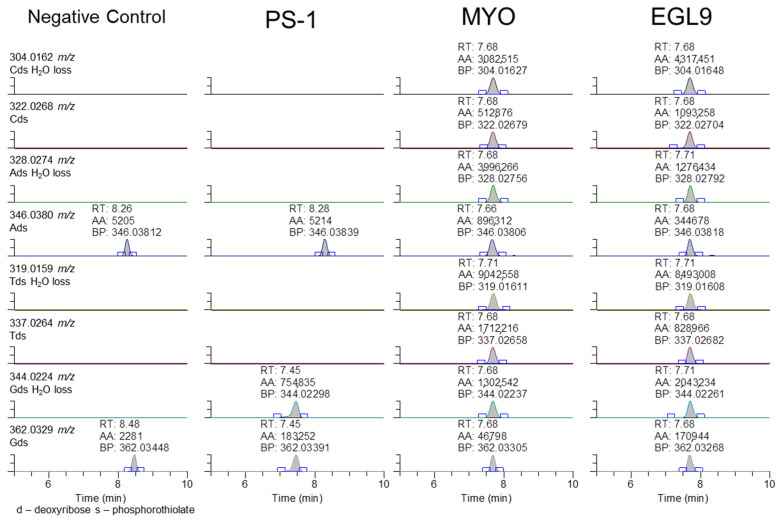
Representative extracted ion chromatograms for PRM targeting 1500 *m*/*z* with 601 *m*/*z* scan window for the negative control serum and serum fortified with the PS-1, MYO, and EGL9 oligonucleotides at the high QC level.

Unfortunately, the presence of a specific fragment associated with the 2′-methoxyethoxy ribose and the phosphorothioate linker could not be observed, suggesting that either the incorrect ions were evaluated or there is further fragmentation that resulted in the losses of ions corresponding to the presence of the 2′-methoxyethoxy modifications. The observation of the product ion containing the phosphorothioate modification and its associated sugar suggested that the addition of these modifications may be observable with their corresponding base. As shown in Figure 8, the phosphorothioate-modified deoxyribonucleotides were observable in the EGL9- and MYO-fortified samples. The presence of the peak at 7.4 min for PS-1 for the 344 and 362 *m*/*z* product ions is attributed to the c1 product ion of the sequence.

### 3.2. Sample Preparation and Extraction

The development of extraction approaches of oligonucleotides from biological matrices is a relatively new area of investigation, with phenol/chloroform liquid–liquid extraction followed by solid-phase extraction being commonly used [18]. Several different extraction approaches were evaluated during the initial method development to determine an optimal approach that balanced obtaining good recoveries, low background, and ease of the procedure with the losses initially observed pre-extraction (omission of the lysis buffer), during evaporation, and in non-specific losses in glassware. The most successful approach involved the utilization of Clarity OTX Solid-Phase Extraction (SPE) cartridges and the corresponding extraction procedure. This method yielded a percent recovery ranging from 56 to 66% and exhibited matrix effect ratios between 0.32 and 0.54 depending on the scan type. 

### 3.3. Limitations

While the development of a non-targeted method capable of detecting phosphorothioated oligonucleotides in equine serum samples opens the possibility for the detection of an entire class of therapeutic agents that was previously non-detectable by methodologies commonly utilized in many drug testing laboratories, there are several limitations of our approach that should be noted. For example, the range of the diversity in the size of synthetic oligonucleotides can be problematic in developing a non-targeted approach. For example, our approach utilizes a molecular weight cutoff filter to concentrate the extract prior to the LC-MS analysis; thus, compounds under 3000 Da are discarded during this concentration step. In addition, the Clarity OTX SPE cartridge was developed to extract oligonucleotides less than or equal to 40 bases long; therefore, the recovery of longer oligonucleotides may not be possible. 

There are several challenges in the implementation of a routine analysis for oligonucleotide-based therapeutic agents in horseracing. The pharmacokinetics of synthetic oligonucleotides are highly variable depending on the specific sequence and targeted matrices, with some agents being quickly eliminated [19]. While much work has been conducted to improve the stability of the exogenous oligonucleotides from digestion by endo-/exonucleases or other losses, oligonucleotide-based therapeutics are susceptible to degradation if appropriate care is not taken during sample collection and transit. One such consideration is through the matrices selected for collection. Many regulatory authorities utilize plasma rather than serum for blood collection, and while not directly assessed in this report, both matrices should be suitable for analysis. As shown for PS-1, extended storage at temperatures warmer than −20 °C results in a dramatic loss. The collection of serum requires the blood to be held at room temperature prior to centrifugation and storage at sub-ambient conditions, while plasma can theoretically be collected, centrifuged, and stored at sub-ambient conditions more quickly, which may reduce the degradation of the oligonucleotides. If coverage for these agents is desired by regulatory authorities, the samples should be transported at sub-ambient temperatures and the analysis initiated as quickly as possible unless the samples are frozen and stored prior to analysis (<−20 °C). 

In our hands, the actual application of the methodology once it is set up can be easily accomplished in a single day, from aliquoting and extraction to an overnight run with a set of 20 samples easily accomplished. Further optimization of the sample extraction approach could enable larger batches of samples to be extracted and analyzed in line with other approaches commonly utilized in equine anti-doping laboratories. Moreover, as the approach utilizes liquid chromatography–mass spectrometry for the detection of the oligonucleotides, the application of this approach can be more easily incorporated into regulatory testing as it would not require ISO-17025-accredited drug testing laboratories to add a completely new test technology to their scope of accreditation. Indeed, there is much bioanalysis of oligonucleotide therapeutic agents supporting either clinical or pre-clinical development, but these are not extraction and LC-MS approaches commonly utilized in racing chemistry laboratories and the successful analysis of these agents requires dedicated methodologies. In our experience, the use of ion-pairing reagents required the use of a dedicated HPLC system, and without this system, switching between mobile phases containing ion-pairing reagents and those more commonly used for bioanalysis such as formic acid makes practical utilization of these approaches more challenging without dedicated equipment. 

While the use of the phosphorothioate-specific product ion provides excellent sensitivity and the ability to detect exogenous administration, it is only specific for the presence of the phosphorothioate bond. The approach of using extracted ion chromatograms to target this ion is unable to differentiate between co-eluting molecules that contain the modification, including metabolites that may not be chromatographically resolved. Moreover, the use of the phosphorothioate-specific product ion makes the incorporation of an internal standard problematic—this approach would not have the ability to differentiate the mass difference between isotopically labeled and unlabeled oligonucleotides, as well as differentiate between unknown and spiked surrogates. Additionally, the use of the extracted ion chromatogram targeting the 94.935 *m*/*z* product ion, following either AIF or PRM acquisition, would not be able to detect non-phosphorothioated oligonucleotides as they would lack the 94.935 *m*/*z* product ion. While the 94.935 *m*/*z* product ion may not be observable in non-phosphorothioated oligonucleotides, the acquisition of non-targeted MS2 spectra using AIF or PRM scan modes opens the possibility for additional exogenous product ions as shown in Figure 5, Figure 6 and Figure 7 to be easily added to processing methods. Additionally, the number of modifications, including the number of phosphorothioate modifications, found in an oligonucleotide are one factor that influences the ability to detect a particular substance, and the reliance on a single modification for identification may reduce the likelihood for detection, especially if the modification is present in low abundance in the oligo. Further, while the MS/MS scan types employed may provide a large amount of product ions following the fragmentation of the targeted molecule using higher-energy C-trap dissociation, full characterization of the exact sequence of nucleotides is a more challenging endeavor without prior knowledge of the various modifications and exact sequence utilized, though work continues to advance in this area [15].

An effective analysis of prohibited substances in horseracing requires a combination of targeted and non-targeted approaches. Targeted approaches can provide high sensitivity and selectivity for substances known to have high potential for abuse but makes the potential detection of new or novel agents highly challenging. Thus, non-targeted approaches for the generation of MS/MS spectra such as using data-independent acquisition (PRM with a large scan window) or triggered data-dependent scans from full-scan acquisitors with or without the use of an inclusion list can offer many advantages, though the resulting data analysis can be quite challenging when conducted in a routine fashion. While there are oligonucleotide-based therapeutics approved for use in humans with many currently in clinical or pre-clinical development, we are unaware of any therapeutics being developed for the horse. Should equine-specific agents be developed or if coverage for oligonucleotide therapeutics developed for humans be desired, the current methodology can be easily adapted for a targeted approach that will likely have a much higher sensitivity and the ability to provide known sequence coverage [9]. In our opinion, the determination of full sequence identity should not be required to report a positive detection as long as sufficient evidence of the presence of exogenous or non-natural modifications is sufficiently established. Further, as the technology surrounding oligonucleotide-based therapeutics advances and their threat to racing safety and integrity increases, it will be crucial to ensure coverage for the modifications used currently and those that will be developed in the future. Accordingly, racing chemistry laboratories will need to simultaneously target the presence of multiple purely exogenous or non-natural combinations of endogenous modifications that occur at low levels to ensure coverage for this class of molecules.

## 4. Materials and Methods

### 4.1. Chemicals, Reagents, and Equipment

Three oligonucleotides (13mer and two 20mers) were purchased from Integrated DNA Technologies and a 16mer oligonucleotide was synthesized at UC Davis (Table 3). For the Beal oligonucleotide, the phosphoramidites were purchased from FireBird Biomolecular Sciences (dZ) (Alachua, FL, USA) and Glen Research (Sterling, VA, USA) (all others) and were incorporated into the 16mer oligonucleotide using an ABI 394 synthesizer. The synthesized oligonucleotide was deprotected with 1 M 1,8-diazabicyclo [5.4.0]undec-7-ene in acetonitrile at room temperature for 10 h, cleaved from solid support with 1:3 ethanol/30% NH_4_OH at 55 °C for 12 h, and desilylated with 55% (*v*/*v*) Et_3_N-3HF at room temperature overnight. The 16mer oligonucleotide was then gel-purified and desalted using a Sephadex G-25 column (Sigma-Aldrich, Burlington, MA, USA), and the mass was confirmed by MALDI-TOF. The 13mer (PS-1) from IDT and the 16mer synthesized by the Beal laboratory did not have specific RNA sequence targets, while the 20mers were custom designed to target EGL9 and myostatin (MYO) gene products in the horse. N,N-diisopropylethylamine (DIEA), hexafluoro-2-propanol (HFIP), nuclease-free water, tetrahydrofuran (THF), and HPLC-grade ethanol were purchased from Sigma Aldrich Inc. The AMICON centrifugal filters (Ultracel^®^-3k) were purchased from VWR. The negative control serum was obtained from BioIVT (Westbury, NY, USA). The Clarity OTX SPE kit containing cartridges and lysis-loading buffer were obtained from Phenomenex. The HPLC-grade water and acetonitrile were obtained from Burdick and Jackson (Muskegon, MI, USA).

### 4.2. Preparation of Reference Standards, Calibrators, and Quality Control Samples

The standards for the oligonucleotides were prepared at a 1 mg/mL concentration in nuclease-free water and diluted to 100, 10, and 1 ng/µL working solutions that were stored at −20 °C until use. A calibration curve was prepared for the PS-1 oligonucleotide at 5, 10, 25, 50, 100, 500, and 1000 ng/mL in the negative control serum. The quality control samples (n = 6/level) were prepared at low (75 ng/mL) and high (750 ng/mL) levels. The QC high level was used to assess the stability at room temperature, 2–8 °C, and −20 °C storage conditions. For the MYO and EGL9 oligonucleotides, the serum was spiked at 50, 100, 250, 500, 1000, and 1500 ng/mL and the quality control samples were prepared at 150 and 1250 ng/mL. Serial dilutions in the serum were used for all the calibrators and quality control samples. The negative controls were run with each set of data. 

### 4.3. Sample Preparation and Solid-Phase Extraction

All the sample preparation steps were performed using 1.5 mL RNase-free microcentrifuge tubes (Thermo Fisher Scientific, Waltham, MA, USA), when possible. The serum (500 µL) was aliquoted followed by the addition of 500 µL of lysis-loading buffer, containing guanidine hydrochloride and Triton X, and the mixture was vortexed and incubated at 4 °C for 15 min. Following the sample pretreatment, the serum was extracted using solid-phase extraction on a Clarity OTX weak anion exchange cartridge (100 mg/3 mL) on a positive pressure extraction manifold. The cartridges were conditioned with 1 mL of methanol and then equilibrated with 1 mL of 50 mM ammonium acetate (pH 5.5), followed by the addition of the pretreated serum samples. After the samples were loaded, the cartridges were washed twice with 3 mL of 50 mM ammonium acetate (pH 5.5) with 50% acetonitrile (ACN). The extract was then eluted from the cartridges with 1 mL of 100 mM ammonium bicarbonate (pH 9.5) with 40% ACN and 10% THF into new microcentrifuge tubes under gravity. The extract was evaporated under N_2_ at 35 °C to approximately 500 µL and was transferred to centrifugal filters and centrifuged for 30 min at 14,000× *g*. The concentrated samples were then transferred to autosampler vials, brought up to volume at 60 µL, capped, and vortexed prior to analysis by LC-MS.

### 4.4. LC-MS Analysis

An instrumental analysis was performed using an Agilent 1100 HPLC coupled to a Thermo Q Exactive HF mass spectrometer (Thermo Fisher Scientific, Waltham, MA, USA). Mobile phase A was composed of HPLC-grade methanol with 15 mM DIEA and 20 mM HFIP. Mobile phase B was composed of HPLC-grade water with 15 mM DIEA and 20 mM HFIP. An ACE C8 (3 µm, 100 × 4.6 mm) held at 35 °C was used as the stationary phase while a reverse phase gradient was used to separate the oligonucleotides with initial conditions of 5%A held for a minute, followed by an increase to 40%A over one minute and to 90% over two minutes, followed by an increase to 98% A over 1.5 min, which was held for 4.5 min prior to column equilibration back to starting conditions for 10 min. Following chromatographic separation, the analytes were introduced to the Q Exactive HF using negative mode electrospray ionization (ESI). The source parameters were as follows: spray voltage of 4500 V, capillary temperature of 350 °C, sheath gas of 60 arbitrary units (au), aux gas of 20 au, spare gas of 2 au, and probe heater temperature of 350 °C. The MS spectra were acquired using 3 different scan modes: all ion fragmentation (AIF), parallel reaction monitoring (PRM), and full-scan MS–selected ion monitoring (SIM). For the AIF scan, the resolution was set at 30,000, with an AGC target of 1 × 10^6^, a collision energy (ce) of 40 eV, a source CID of 60 eV, and a scan range of 90–1350 *m*/*z*. For the PRM acquisition, with an in-source CID of 0 eV, the resolution was set at 60,000, with an AGC target of 2 × 10^5^, a loop count of 1, a default charge state of 8, an isolation window of 601 *m*/*z*, a fixed first mass of 90 *m*/*z*, and a stepped ce of 20 and 40 eV. The inclusion list for the PRM was 900 *m*/*z* with a default charge state of 5, 1500 *m*/*z* with a default charge state of 3, and 2100 *m*/*z* with a default charge state of 3. For the full MS–SIM acquisition, it had an in-source CID of 0 eV, the resolution set at 60,000, an AGC target of 3 × 10^6^, and a scan range of 600–6000 *m*/*z*. The data collected were stored using the Xcalibur 4.3 data system and processed using the Thermo Qual browser and Quan browser.

### 4.5. Method Validation

The method was validated to assess the linearity, limits of detection, limits of quantitation, accuracy (% difference), precision (RSD), stability (% expected), recovery, and matrix effects for the PS-1 oligonucleotide over multiple days. The limit of detection was defined as the smallest concentration assessed that was indistinguishable from baseline (signal:noise > 10:1). The limits of quantification were determined for each scan type as the amount with less than 30% variability from the theoretical concentration. The matrix effects were calculated as a ratio of the peak area response from a post-extraction spiked sample as compared to the corresponding neat standard at the high QC level. The percent recovery was calculated using the peak area for an extracted sample compared to the peak area of a post-extraction spiked sample at the high QC level. The method was further evaluated for accuracy, precision, and limits of detection for the EGL9 and MYO oligonucleotide sequences.

## 5. Conclusions

A liquid chromatography–high-resolution mass spectrometry method was developed and validated that can successfully detect phosphorothioated oligonucleotides in equine serum. The methodology shows the feasibility of using this method to detect unknown phosphorothioated oligonucleotides as part of a screening method for a large entire class of therapeutic agents that was previously non-detectable by methodologies commonly utilized in most drug testing laboratories.

## Figures and Tables

**Table 1:** Inter- and intra-day accuracy and precision PS-1.

		QC Low (75 ng/mL)		QC High (750 ng/mL)	
	Scan Type	Percent Difference	RSD	Percent Difference	RSD
Day 1	PRM-900 *m*/*z*	39.6%	5.9%	15.5%	12.4%
	PRM-1500 *m*/*z*	46.6%	8.0%	58.7%	11.7%
	PRM-2100 *m*/*z*	37.6%	7.9%	50.8%	11.9%
	AIF	27.7%	5.0%	16.3%	12.3%
Day 2	PRM-900 *m*/*z*	17.9%	15.7%	1.1%	13.3%
	PRM-1500 *m*/*z*	24.1%	8.4%	9.7%	17.9%
	PRM-2100 *m*/*z*	17.4%	9.6%	4.4%	18.0%
	AIF	22.5%	10.6%	2.0%	12.9%
Day 3	PRM-900 *m*/*z*	37.0%	18.3%	10.2%	7.0%
	PRM-1500 *m*/*z*	31.1%	14.8%	11.1%	9.5%
	PRM-2100 *m*/*z*	30.9%	14.9%	12.0%	6.0%
	AIF	30.2%	10.5%	11.0%	7.0%
Inter-Day	PRM-900 *m*/*z*	31.5%	20.1%	2.1%	15.3%
	PRM-1500 *m*/*z*	34.0%	18.0%	12.6%	32.3%
	PRM-2100 *m*/*z*	28.6%	16.1%	11.5%	28.6%
	AIF	26.8%	9.7%	1.1%	15.8%

**Table 2:** PS-1 stability at various storage conditions.

Scan Type	24 h at ~25 °C	24 h at 4 °C	27 Days at 4 °C	27 Days at −20 °C	28 Days at −20 °C 1× Frz/Thaw	28 Days at −20 °C 2× Frz/Thaw
PRM-900 *m*/*z*	ND	51.7%	4.0%	61.1%	44.3%	53.0%
PRM-1500 *m*/*z*	26.9%	51.5%	4.7%	55.9%	50.3%	55.5%
PRM-2100 *m*/*z*	ND	62.8%	5.1%	53.9%	57.4%	65.0%
AIF	28.5%	54.4%	3.9%	59.9%	48.7%	54.8%

**Table 3:** Targeted oligonucleotide sequences.

Base Length	Name	PS Modified	Sequence
13	PS-1	12	5′–rA* rU* rC* rA* rG* rG* rU* rC* rA* rC* rU* rG* rC–3′
20	HorseMyostatin (MYO)	19	/52MOErC/*/i2MOErT/*/i2MOErT/* /i2MOErC/*/i2MOErA/*C* A*T*C* A*A*T* G*C*T* /i2MOErC/*/i2MOErT/*/i2MOErG/*/i2MOErC/*/32MOErC/
20	EGL9	19	/52MOErT/*/i2MOErT/*/i2MOErA/* /i2MOErC/*/i2MOErC/*T* T*G*G* C*A*T* C*C*C* /i2MOErA/*/i2MOErG/*/i2MOErT/*/i2MOErC/*/32MOErT/
16	Beal	4	mC*mG*mArCrCrCrGrCdZdNrArUrUmC*mU*mC

Abbreviation: r—Ribose, 5mC—5 methyl Cytidine, m—2′-O-Methyl, d—2′-Deoxy, *—Phosphorothioate modification, A—Adenosine, G—Guanosine, C—Cytidine, T—Thymidine, U—Uridine, 52MOErC—5′ 2′-MethoxyEthoxy rC, 52MOErT—5′ 2′-MethoxyEthoxy rT, i2MOErT—Internal 2′-MethoxyEthoxy rT, i2MOErC—Internal 2′-MethoxyEthoxy r5mC, i2MOErA—Internal 2′-MethoxyEthoxy rA, i2MOErG—Internal 2′-MethoxyEthoxy rG, 32MOErC—3′ 2-MethoxyEthoxy r5mC, 32MOErT—3′ 2-MethoxyEthoxy rT, dZ—6-amino-5-nitro-3-(1′-β-D-2′-deoxyribofuranosyl)-2(1H)-pyridone, and N—8-azanebularine.

## Data Availability

The data presented in this study are available upon reasonable request from the corresponding author.
